# LINE-1 Cargo and Reverse Transcriptase Activity Profiles in Extracellular Vesicles from Lung Cancer Cells and Human Plasma

**DOI:** 10.3390/ijms23073461

**Published:** 2022-03-22

**Authors:** Emma C. Bowers, Alexandre Motta, Ken Knox, Brian S. McKay, Kenneth S. Ramos

**Affiliations:** 1 Center for Genomic and Precision Medicine, Texas A&M Institute of Biosciences and Technology, Houston, TX 77030, USA; emma.ciel@gmail.com; 2 University of Arizona College of Medicine-Tucson, Tucson, AZ 85724, USA; cavalcante@email.arizona.edu; 3 Department of Internal Medicine, Division of Pulmonary Medicine, University of Arizona College of Medicine-Phoenix, Phoenix, AZ 85004, USA; kknox@deptofmed.arizona.edu; 4 Department of Ophthalmology and Vision Science, University of Arizona College of Medicine-Tucson, Tucson, AZ 85724, USA; bmckay@eyes.arizona.edu

**Keywords:** LINE-1, extracellular vesicles, exosomes, benzo[a]pyrene, BaP, reverse transcriptase, lung cancer, liquid biopsy, ORF1p, ORF2p, retrotransposition, retrotransposons

## Abstract

Long Interspersed Element-1 (LINE-1) is an oncogenic human retrotransposon that ‘copies and pastes’ DNA into new locations via reverse transcription. Given that enzymatically active LINE-1 can be exported in extracellular vesicles (EVs), and that LINE-1 mRNA and its two encoded proteins, ORF1p and ORF2p, are required for retrotransposition, the present study examined LINE-1 EV loading patterns relative to reverse transcriptase (RT) activity in vivo and in vitro. Density gradient ultracentrifugation identified conserved patterns of LINE-1 mRNA and protein distribution in EVs, with RT activity readily detected in EV fractions containing both LINE-1 mRNA and protein. Unlike whole cell and tissue lysates, the ORF1p in EVs was detected as a dimer. EVs from ostensibly healthy plasma donors showed variable but consistent ORF1p profiles, with residual levels of LINE-1 mRNA measured in some but not all samples. EVs from cancer cell lines had elevated mean LINE-1 levels and 5–85 times greater RT activity than EVs from normal cells or healthy plasma. EV RT activity was associated with EV LINE-1 mRNA content and was highest in cell lines that also expressed an elevated expression of ORF1p and ORF2p. Given that LINE-1 activation is a hallmark of many cancer types, our findings suggest that an EV LINE-1 ‘liquid biopsy’ may be developed to monitor LINE-1 activity during the course of malignant progression.

## 1. Introduction

**Figure 1:**
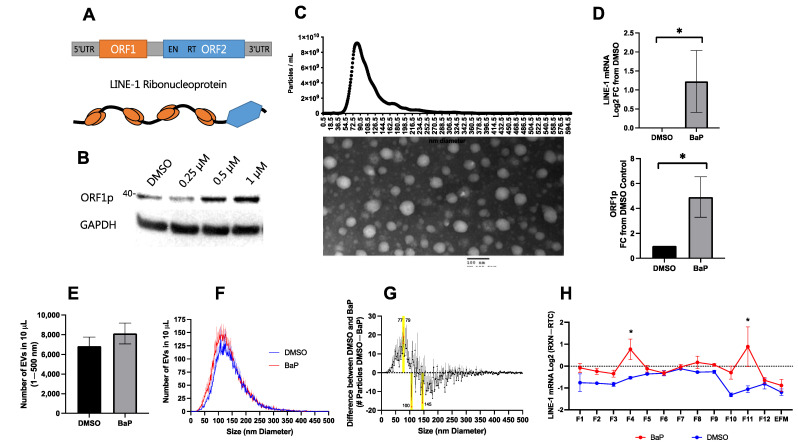
Long Interspersed Element-1 (LINE-1) induction by benzo[a]pyrene and its impact on extracellular vesicle (EV) LINE-1 content, number, and size. (**A**) LINE-1 element domain structure. LINE-1 is a 6 kb repetitive DNA element that comprises 5′ and 3′ untranslated regions (UTRs) and two proteins, ORF1p and ORF2p. ORF1p is a 40 kDa nucleic acid binding protein that forms multimers. ORF2p is 150 kDa and contains both endonuclease (EN) and reverse transcriptase (RT) domains. ORF1p trimers and ORF2p exhibit strong affinity for the LINE-1 mRNA, and together form a ribonucleoprotein (RNP) containing both the template and enzymatic machinery required for target-site primed reverse transcription. (**B**) ORF1p induction in H460 cells by benzo[a]pyrene (BaP). To examine LINE-1 content in EVs, we used a LINE-1 inducer, the carcinogen BaP. (**C**) EV preparation. EVs were collected from cells challenged with 1 μM BaP for 48 h. The size and morphology of the preparation was assessed using Nanosight Nanoparticle Tracking Analysis (NTA) (top) and electron microscopy (bottom). (**D**) H460 EV LINE-1 content. BaP exposure increased EV LINE-1 mRNA and ORF1p content in BaP treatments compared to DMSO controls (as measured by One-Step RT-qPCR and ELISA; * *p* < 0.05; Welch’s *t*-test) but did not significantly alter the total abundance of EVs (*p* > 0.05; *t*-test), as shown in (**E**). NTA of BaP and DMSO treatments (**F**). (**G**) BaP-induced size differences. To examine subtle changes in EV size, total EV numbers were normalized to 5000 particles per reading, and each EV size bin was compared between treatments using a two-way ANOVA. Yellow bars indicated EV sizes with significant differences. *N* = 3–5. (**H**) Test gradient (BaP vs. DMSO control). For subsequent density gradient EV studies in this cell line, we performed a test gradient and determined that BaP exposure (red) was necessary to raise LINE-1 expression to detectable levels. * Difference between DMSO and BaP (*p* < 0.05; two-way ANOVA).

Up to 20% of the human genome consists of LINE-1 (Long Interspersed Element-1) sequences. LINE-1 is a ~6 kB DNA retroelement that ‘copies and pastes’ itself and other DNAs into different locations throughout the genome. While most LINE-1s are truncated sequences devoid of functional activity, a diploid human genome contains ~100 full-length LINE-1 elements capable of full cycles of retrotransposition [1,2]. Their activity is regulated by epigenetic silencing through mechanisms that involve DNA methylation and covalent histone modifications [3]. Upon removal of silencing epigenetic marks, LINE-1 is transcribed from two open reading frames (ORFs) and translated into two proteins, ORF1p and ORF2p (Figure 1A). ORF1p is a coiled-coil protein with chaperoning activity, while ORF2p is a protein with endonuclease and reverse transcriptase (RT) activities. Compared to ORF1p, ORF2p is expressed in sub-stoichiometric amounts and has highly restricted translation in most cell types [4,5,6]. These proteins have strong affinity for the LINE-1 mRNA and form ribonucleoprotein (RNP) particles that contain both the template and the machinery necessary to initiate retrotransposition.

The activation of LINE-1 occurs in different biological contexts. First, LINE-1 can be activated by DNA damage or agents that perturb the epigenome, as seen during inflammation, oxidative stress, or toxic injury [7,8]. Second, as the genome of cancer cells becomes unstable, epigenetic control of LINE-1 is eroded and expression markedly enhanced [9,10]. LINE-1 oncogenesis is mediated by the insertion of mutations or deletions, genome reprogramming [11,12,13]; and/or activation of oncogenic signaling independent of retrotransposition [14].

Given the dual role of LINE-1 as an effector and a target of oncogenic changes, measures of LINE-1 activation can serve as cancer biomarkers. LINE-1 DNA hypomethylation is a common feature of many cancers [15], and there are strong correlations between LINE-1 readouts and cancer status, tumor instability, and cancer mortality [9,10,13,15,16]. Although meaningful, these approaches are often limited in that they depend on invasive methodology and are not readily adaptable for widespread screening. To circumvent these limitations, LINE-1 cargo and/or enzymatic activity within extracellular vesicles (EVs) could, in principle, be used as a proxy of cellular LINE-1 activity. While the presence of enzymatically active LINE-1 products in EVs has been documented [17,18], these observations have been limited to ectopic expression models and/or several cancer cell lines. Thus, far too little is currently known about EV LINE-1 to determine its suitability as a “liquid biopsy” [19].

To this end, we examined the EV loading of LINE-1 mRNA and its proteins and their relationship with RT activity. In our study, we used EVs from cell cultures under both constitutive and induced conditions or plasma from ostensibly healthy donors. In addition, One-Step RT-qPCR was employed to enhance the sensitivity of our qPCR assays. Using buoyant density gradient ultracentrifugation, we discovered conserved EV LINE-1 loading patterns, with some EV populations containing only LINE-1 mRNA, while others contained both LINE-1 mRNA and protein. RT activity was present in fractions containing both LINE-1 mRNA and protein, supporting the notion that LINE-1 RNP formation enables enzymatic activity. An ORF1p dimer was enriched in EVs, whereas ORF1p in monomeric form was predominantly seen in cell lysates. An analysis of EV LINE-1 in human plasma donors revealed a variable but consistent presence of ORF1p and residual levels of EV LINE-1 mRNA in several donors. While healthy donors and non-cancerous cells had residual RT activity levels, lung cancer cell lines exhibited higher mean LINE-1 EV content and RT activity. RT activity was significantly associated with EV LINE-1 mRNA content and was highest in cells expressing both ORF1p and ORF2p. Taken together, these results provide a unique insight into the development of EV-based LINE-1 bioassays for investigational and clinical applications.

## 2. Results

### 2.1. LINE-1 Induction by benzo[a]pyrene (BaP) to Study the Loading of LINE-1 in EVs

**Figure 2:**
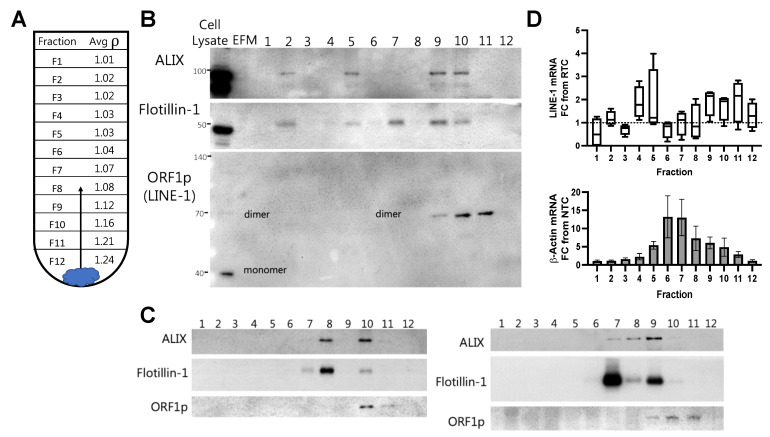
Buoyant density gradient ultracentrifugation demonstrates the presence of Long Interspersed Element-1(LINE-1) mRNA and ORF1p in high- and low-density extracellular vesicles (EVs). EVs from H460 cells stimulated with 1 μM BaP were subjected to iodixanol buoyant density gradient ultracentrifugation to examine the presence of LINE-1 mRNA and protein within EVs and their distribution across EV populations. (**A**) Mean densities of 1 mL fractions from three gradient preparations. Diagram showing how EVs rise to the gradient level equivalent to their density during the course of centrifugation. (**B**) Western blot of EV proteins present in density gradient fractions 1–12. ALIX and Flotillin-1 are proteins enriched in EVs. ORF1p, which often appears in multimers in SDS-PAGE, is visible as a putative dimer within EVs. Cell lysate is shown as a positive control, EV-free media (EFM) as a negative control. (**C**) Western blots from two additional independent gradients. (**D**) mRNA in gradient fractions. mRNA was quantified by One-Step RT-qPCR. Amplicons were expressed as fold-change from the Reverse Transcriptase Control (LINE-1, top) or Non-Template Control (β-Actin, bottom). For LINE-1, the minimum, maximum, and mean are shown in box plot. β-Actin, mean and SEM shown. *N* = 3–4.

BaP activates LINE-1 in both normal and cancer lung epithelial cells [20,21,22] (Figure 1B). Using H460 lung cancer cells, we first examined the impact of BaP exposure on the EV loading of LINE-1. Cells were challenged with DMSO (vehicle) or 1 μM BaP and allowed to condition the media for 48 h before the isolation of EVs. Following PEG precipitation, isolates were confirmed by electron microscopy to contain EVs and to be free of debris by nanoparticle tracking analysis (NTA), as shown in the top panel of Figure 1C. The presence of EV marker proteins is detailed in Figure 2. EV ORF1p levels were detected by ELISA, as residual PEG is known to interfere with SDS-PAGE. LINE-1 mRNA was detected by One-step RT-qPCR, which enables sensitive detection of less abundant transcripts. Because LINE-1 contains no introns that distinguish between genomic DNA and mRNA-derived cDNA, each LINE-1 sample was normalized to a matched control lacking RT (RTC) to control for any residual gDNA contamination. LINE-1 mRNA increased by an average of 2.33-fold and ORF1p levels increased 4.92-fold relative to the DMSO controls (Welch’s *t*-test; *p* < 0.05).

To ascertain whether BaP altered EV secretion, we examined total EV number and size using NTA. Given the relative absence of particles above 500 nm in our preparations (Figure 1C), this size cut-off was used for particle analysis. While the mean number of EVs modestly increased from 6839 to 8123 particles as a result of BaP treatment, this difference was not significant (Figure 1E,F). Proportional scaling of EVs to 5000 particles ranging in size from 1 to 500 nm showed that BaP slightly increased the secretion of smaller size particles < 130 nm and inhibited the release of larger size particles from 140 to 250 nm (Figure 1G). Statistically significant increases were seen in EVs of 77 and 79 nm, while EVs with diameters between 107 and 145 nm significantly decreased (two-way ANOVA; *p* < 0.05).

In subsequent studies, buoyant density gradient ultracentrifugation was used (Figure 2) to assess LINE-1 mRNA detection with and without BaP treatment (Figure 1H). We discovered that basal H460 LINE-1 mRNA in EVs was relatively low and that BaP stimulation was necessary to enhance LINE-1 mRNA detection (two-way ANOVA; *p* < 0.05). Together these results demonstrate that the LINE-1 inducer, BaP, can be effectively used to study the packaging of LINE-1 within EVs.

### 2.2. LINE-1 EV Cargo Distribution in BaP-Stimulated Cells

To further examine the presence of LINE-1 in EVs and its loading patterns, EV pellets were placed on the bottom of the ultracentrifugation tube and allowed to rise to a gradient layer equivalent to their density, thus leaving free protein and debris behind. The density of exosomes and microvesicles ranges from 1.05–1.21 g/mL; however, small EVs can have densities as low as ~1.03 g/mL [23]. The average density of 1 mL fractions from three different gradients is shown in Figure 2A. For Western blotting (Figure 2B), EV-free medium (EFM) was used as a negative control, while total cell lysate was used as a reference control. ALIX and Flotillin-1, proteins enriched in many EVs [24], were most abundant in Fractions 7–11 (~1.07–1.21 g/mL), with bands of variable intensity seen across Fractions 2–6 (1.02–1.04 g/mL). In keeping with the ability of ORF1p to form multimers that are resistant to denaturation under reducing SDS-PAGE conditions, a 70 kDa band was detected in EVs that we and others have identified as a putative ORF1p homodimer [25,26]. However, the possibility remains that the 70 kDa band represents a denaturation-resistant heterodimer of ORF1p with another cellular protein. In either case, the 70 kDa dimer contrasts with the 40 kDa monomeric species typically detected in cell lysates. The 70 kDa band was detected in Fractions 9–11, with the highest levels consistently appearing in Fractions 10 and 11 and corresponding to EVs with densities approximating ~1.12–1.21 g/mL. Two additional gradients are shown in Figure 2C.

Gradient fractions were then assessed for LINE-1 mRNA content (Figure 2D, top). β-Actin is shown as a reference (Figure 2D, bottom). The abundance of LINE-1 mRNA was variable across fractions. A LINE-1 mRNA peak was typically observed in Fractions 4–5, usually corresponding to EVs with a density of ~1.03 g/mL, with a second peak often occurring around Fractions 9–11 with average densities of ~1.12–1.21 g/mL These results demonstrate that LINE-1 mRNA and ORF1p are present within EVs, and suggest that a cargo loading program produces at least two distinct types of LINE-1 EV populations: those containing only LINE-1 mRNA (~1.03 g/mL), and those containing both LINE-1 mRNA and ORF1p (~1.12–1.21 g/mL).

### 2.3. Characterization of LINE-1-Containing Vesicles

**Figure 3:**
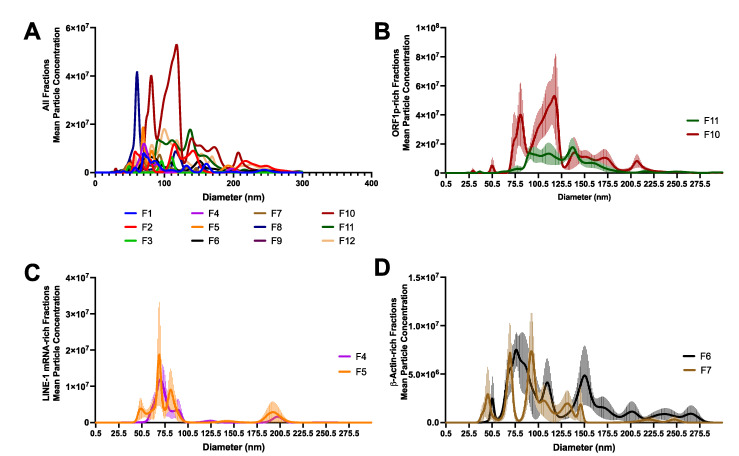
Characterization of Long Interspersed Element-1 (LINE-1)-containing extracellular vesicles (EVs). We examined EVs from the density gradient fractions shown in Figure 2 using Nanosight Nanoparticle tracking analysis (NTA). (**A**) NTA profiles of all fractions. (**B**) ORF1p-rich fractions. ORF1p was consistently found in EVs with a density greater than ~1.12 g/mL, most often in Fractions 10 and 11 (~1.16–1.2 g/mL). (**C**) LINE-1 mRNA-rich fractions. LINE-1 mRNA was detected in EVs with low-to-moderate density (~1.03–1.07). (**D**) β-Actin-rich fractions. Moderate-density fractions with the highest enrichment for β-Actin are shown for comparison. Mean of five readings ± SE shown.

Nanosight NTA was used to examine the size profiles of the density fractions described in Figure 2. The size and particle concentrations of all fractions are depicted in Figure 3A. Fractions 10 and 11 (Figure 3B) were enriched with ORF1p and to a lesser extent LINE-1 mRNA. Fraction 10 contained two peaks, one near 75.5 nm and another broad peak around 120 nm, the latter containing a broad shoulder of larger particles tapering off around ~215 nm. Fraction 11 had fewer vesicles of broad distribution ranging from 80 to 180 nm. Fractions 4 and 5 containing LINE-1 mRNA had a narrow size range from 50 to 100 nm, with a punctate peak around 75 nm (Figure 3C). Medium-density fractions containing no/minimal LINE- but abundant β-Actin exhibited peaks of many different sizes ranging from 50 to 275 nm. These results suggest that EVs containing only LINE-1 mRNA are likely small (~75 nm), low-density EVs, while EVs containing ORF1p are likely larger size, high-density vesicles.

### 2.4. LINE-1 EV Cargo Distribution in Human Plasma

**Figure 4:**
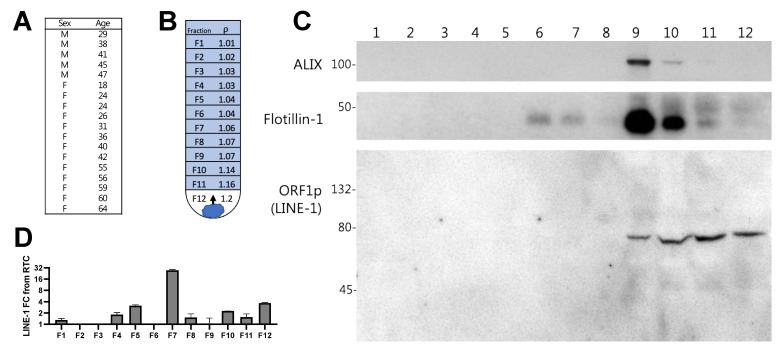
Density gradient distribution of Long Interspersed Element-1 (LINE-1) mRNA and ORF1p in human plasma extracellular vesicles (EVs). Plasma EVs from ostensibly healthy subjects were isolated by ultracentrifugation and subjected to iodixanol buoyant density gradient ultracentrifugation. Plasma from 18 subjects (1 mL each) was pooled to increase likelihood of LINE-1 detection (**A**) Age and sex of donors. (**B**) Diagram showing how EVs rise to the gradient level equivalent to their density during the course of centrifugation. Density of 1 mL gradient fractions. (**C**) Western blot of EV proteins and the ORF1p dimer present in density gradient fractions 1–12. (**D**) LINE-1 mRNA present in fractions 1–12.

Next, we sought to determine whether the LINE-1 distribution patterns described in vitro were upheld in vivo in human plasma. One milliliter of plasma from 18 ostensibly healthy human subjects was pooled to improve the detection of LINE-1 (Figure 4A). EVs were subjected to buoyant density gradient ultracentrifugation as described, with fraction densities shown in Figure 4B. Western blotting (Figure 4C) demonstrated that in human plasma EVs, as seen in vitro, the ORF1p dimer was predominant and detected in Fractions 9–12, peaking in Fractions 10 and 11 (~1.14–1.16 g/mL). At least a two-fold enrichment of LINE-1 mRNA was detected in Fractions 5, 7, 10, and 12, with the highest level of detection, a 32-fold enrichment from the RTC, seen in Fraction 7 (~1.06 g/mL) (Figure 4D). These results indicate that LINE-1 mRNA and ORF1p are present in plasma EVs, and exhibit similar loading patterns to EVs derived from BaP-stimulated cells.

### 2.5. EV LINE-1 Content in Individual Plasma Donors

**Figure 5:**
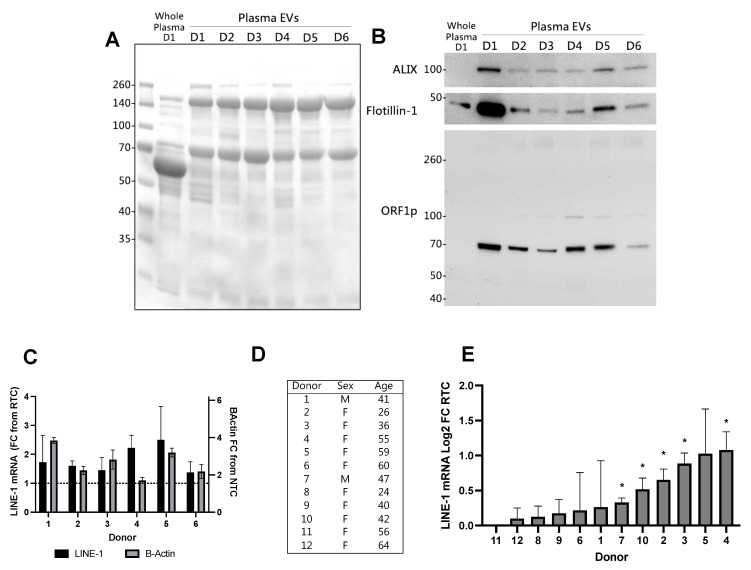
Extracellular vesicles (EV) Long Interspersed Element-1 (LINE-1) levels in individual human plasma donors. LINE-1 mRNA and ORF1p levels were quantified in ostensibly healthy donors using plasma volumes that could be commonly acquired by venipuncture. For each donor, EVs were isolated from 2.5 mL plasma via ultracentrifugation. Thirty micrograms of EV protein from six different donors (D1–D6) was used for Western blotting. (**A**) Ponceau staining of the membrane demonstrates equal protein loading. Whole plasma from Donor 1 was included as a reference. (**B**) Immunoblot of EV proteins and ORF1p. (**C**) RNA was extracted from EVs and then subjected to One-Step RT-qPCR. Mean, SEM shown. To examine LINE-1 mRNA expression more closely, mRNA levels from all donors used in this study were converted to log2 FC values (**D**,**E**) and a one-sample *t*-test was used to determine if levels were significantly greater than zero (* *p* < 0.05).

The EV LINE-1 content of individual plasma donors was then examined using plasma volumes commonly acquired in clinical or research settings. Platelets were removed from 2.5 mL plasma-EDTA and EVs were isolated by ultracentrifugation. Half of the EV preparation was used for Western blotting and the remainder for LINE-1 mRNA quantification by One-Step RT-qPCR. Thirty micrograms of EV protein was subjected to SDS-PAGE. Figure 5A shows the total protein Ponceau stain, with whole plasma from Donor 1 shown as a reference. All EV preparations were positive for the EV markers ALIX and Flotillin-1 (Figure 5B), although significant variation in enrichment was observed. The ORF1p dimer was predominant in EV fractions, with some donors showing evidence of trimeric ORF1p. Whole plasma (Lane 1) did not show detectable ORF1p. Variable enrichment in ORF1p was seen in EVs from different donors: high in Donor 1 but only faintly detectable in Donor 6.

We then measured EV LINE-1 mRNA in the same donors (Figure 5C). Although β-Actin was detected in EVs from all donors, LINE-1 mRNA was only present in trace amounts. In order to ascertain whether these readings were experimental ‘noise’ or positive, albeit at low-levels of detection, the FC values were log2 converted for a one-sample *t*-test analysis compared to no detection. We performed this analysis using 12 donors from Figure 5A–C and 8 (Figure 5D). LINE-1 mRNA detection levels ranged from −0.04 to 1.03 log2 FC from RTC (Figure 5E). Of these 12 donors, five exhibited significant LINE-1 mRNA detection.

### 2.6. Quantifying RT Activity in H460 EVs and Plasma Donors

**Figure 6:**
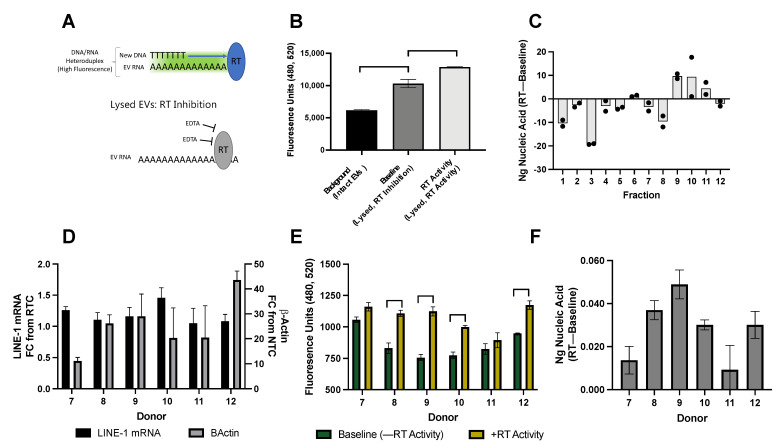
Reverse transcriptase activity in EVs from BaP stimulated cells and healthy plasma donors. (**A**) The modified ENZ-check reverse transcriptase (RT) activity assay indicates the presence of EV-derived reverse transcriptase (RT) and its ability to use extracellular vesicle (EV) mRNA and DNA as a template. During the reaction, any reverse transcriptase enzyme (i.e., ORF2p) will use EV mRNA and DNA to form RNA-DNA heteroduplexes, which bind PicoGreen and emit a fluorescent signal. This RT signal is normalized to baseline fluorescence emitted by EV DNA cargo, which is measured by inhibiting RT activity with EDTA. EVs were collected by ultracentrifugation. (**B**) Determination of baseline fluorescence and RT activity. Raw fluorescence of RT activity, background, and baseline conditions. “Background, Intact EVs” reaction constituents and PicoGreen are prevented from accessing EV cargo as the EV lipid membrane is still intact. “Baseline, RT Inhibition” EVs are lysed allowing PicoGreen to bind with DNA cargo but EDTA prevents RT activity. “RT Activity” EVs were lysed and conditions permissive for RT activity. One-way ANOVA: *p* < 0.05. (**C**) RT activity in EVs collected from BaP-treated cells. Fractions from Figure 2 were examined for RT activity and a DNA standard curve was used to quantify the amount of new nucleic acid generated in the RT reaction. To detect RT activity in individual plasma donors, EVs were isolated from 2.5 mL plasma via ultracentrifugation. Half the preparation was used for mRNA quantification and the other for RT activity measurement. The age and sex of plasma donors are shown in Figure 5D. (**D**) EV Long Interspersed Element-1 (LINE-1) mRNA and β-Actin levels. (**E**) Nucleic acid fluorescence. RT activity assay showing raw fluorescence values of baseline and RT activity conditions. *p* < 0.05: two-way ANOVA with Sidak’s multiple comparisons. (**F**) EV RT activity measurement in ng of nucleic acid. Mean, SD shown.

Previous studies have demonstrated that EV LINE-1 is capable of carrying out retrotransposition in recipient cells and modifying their DNA [17,18]. We expanded these findings by further characterizing EV RT activity in vitro and in vivo (Figure 6). LINE-1 reverse transcription is mediated by the activity of ORF2p, which initiates target-site primed reverse transcription (TPRT) by nicking genomic DNA to create a 3′-OH, which serves as a primer for LINE-1 to reverse transcribe its mRNA. We used a modified activity assay to measure TPRT by quantifying the formation of RNA-DNA heteroduplexes that fluoresce in the presence of PicoGreen (Figure 6A).

First, we validated this assay using several controls. “Background” demonstrates fluorescent signal from intact EVs in which PicoGreen is prevented from interacting with EV contents by the EV lipid membrane. To control for baseline genomic DNA levels within EVs once they are lysed, each RT-competent reaction (“RT Activity) was normalized to a control reaction in which RT activity was inhibited with EDTA (“Baseline”).

Figure 6B demonstrates the fluorescence of EVs isolated from BaP-stimulated H460 cells under the various aforementioned conditions. The mean (±SD) fluorescence of intact EVs was 6059 ± 149 units, which increased to 10,327 ± 637.1 units in lysed EVs treated with EDTA inhibitor. The signal intensity further increased to 12,859 ± 37.5 units when the lysed EVs were allowed to undergo reverse transcription in the absence of EDTA. Thus, our assay was sufficiently sensitive to detect RT activity in EVs isolated from BaP-treated cells.

In light of the LINE-1 distribution patterns presented in Figure 1 and Figure 3, we first sought to determine if individual fractions were enzymatically active. The presence of RT activity within a fraction implies that all the necessary equipment for reverse transcription (i.e., LINE-1 mRNA, ORF1, and ORF2) is present within the same fraction. The RT activity of each fraction was calculated as previously described and a DNA standard was used to calculate the amount of nucleic acid generated from the RT reaction (Figure 6C). We found that the nucleic acid content was unchanged or degraded in Fractions 1–8 and Fraction 12 over the course of the reaction, whereas new nucleic acid was generated in Fractions 9, 10, and 11. Although ORF2p was not detectable in individual fractions by Western blotting, these results suggest that ORF2p is exported within these enzymatically-active fractions.

Next, we questioned whether RT activity could be detected in plasma EVs from individual donors (Figure 6D–F). EVs from 2.5 mL plasma (donors in Figure 5D) were collected by ultracentrifugation. Half the preparation was used to quantify LINE-1 mRNA (Figure 6D) and the other to measure RT activity, as previously described. β-Actin was detected in all samples and LINE-1 mRNA ranged from 1.05 to 1.46 FC from RTC. The raw fluorescence of baseline-lysed EVs and RT-permissive reactions (Figure 6E) ranged between 732 and 1207 fluorescence units, with some donors exhibiting increases in fluorescence as a result of RT activity. The amount of nucleic acid generated during the RT reactions ranged from 0.009 to 0.049 ng (Figure 6F).

### 2.7. EV RT Activity in Lung Cancer Cell Lines and Comparison with LINE-1 Levels

**Figure 7:**
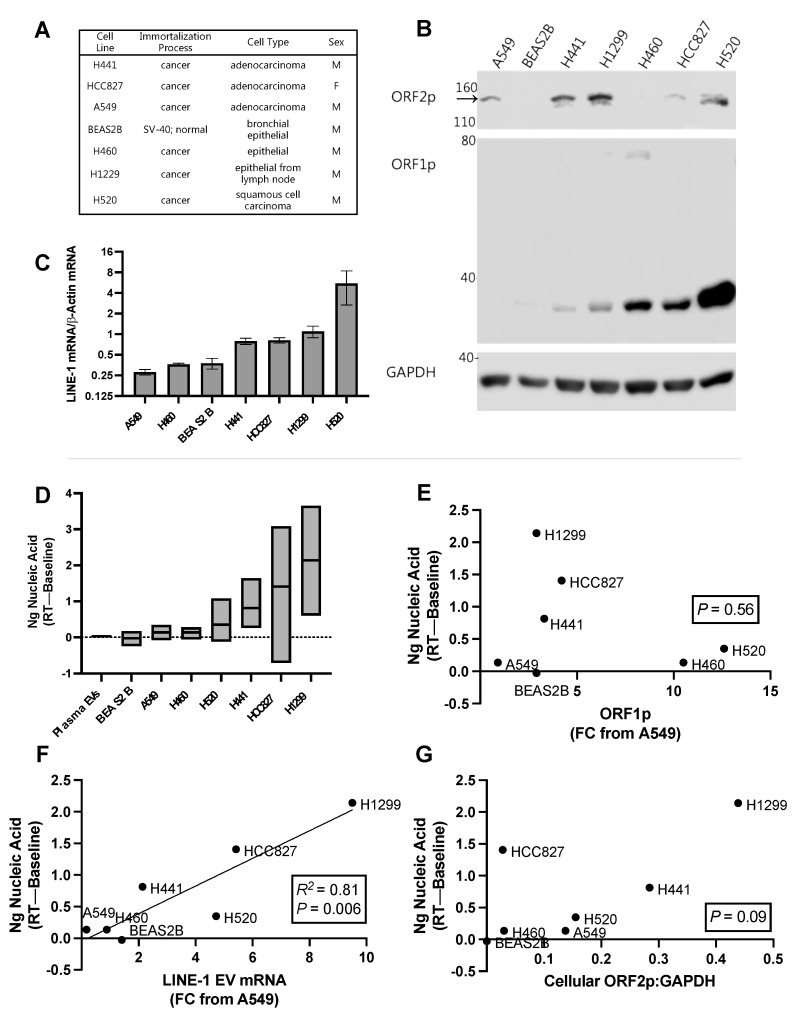
Relationship between Long Interspersed Element-1 (LINE-1) components and reverse transcriptase activity (RT) in extracellular vesicles (EVs) from lung cancer cell lines. (**A**) Characteristics of one normal (BEAS2B) and six cancer cell lines used in the experiment. (**B**) Cellular ORF1p and ORF2p expression. Representative Western blot using 75 μg protein depicting quantities of cellular ORF1p and ORF2p. The ORF2p band is the top band, indicated by the black arrow. (**C**) Quantification of cellular LINE-1 mRNA levels using One-Step RT-qPCR (Mean, SEM shown). (**D**) EVs from three independent cultures and EV collections were measured for RT activity (min, max, and mean shown). RT activity from the plasma donors in 6F are shown for reference (“Plasma EVs”). We then quantified LINE-1 components in EVs using an ORF1p ELISA and One-Step RT-qPCR. These measures were normalized to A549s, which exhibited the lowest levels of ORF1p and LINE-1 mRNA. As LINE-1 RT activity requires the formation of a ribonucleoprotein comprised of its proteins and mRNA, we compared RT activity to the levels of each of these components to determine if one of them could be used as a predictor of RT activity (R^2^ and *p* levels shown in each figure (**E**,**F**). As ORF2p in EV isolates was not detected by Western blotting, we compared EV RT activity with mean cellular ORF2p levels, as approximated by the densitometric analysis of ORF2p Western blots (**G**). *N* = 3.

Next, we used a panel of cell lines to investigate how EV LINE-1 levels influence RT activity (Figure 7A). For this experiment we selected lung cell lines, as LINE-1 activation markers are closely associated with increased lung cancer risk, pathological features, and clinical outcomes [9,10,27]. Endogenous cellular LINE-1 protein expression from the cell panel is depicted in Figure 7B. By loading a relatively large amount of protein (75 μg) we detected ORF2p by Western blotting in most cell lines (top band; black arrow), but not BEAS-2B (the non-cancer cell line) and H460. ORF1p, with higher levels of endogenous expression, is shown for comparison. The non-cancer cell line BEAS-2B exhibited the lowest overall LINE-1 protein levels, with undetectable ORF2p and faint ORF1p. ORF1p was not detected in A549 cells, but a relatively strong ORF2p band was observed. H1299 cells exhibited the highest ORF2p expression levels, along with moderate levels of ORF1p. Conversely, H520 cells exhibited the highest ORF1p expression levels with moderate ORF2p. Note that the predominant form of ORF1p in cell lysates is the monomer, but that a light band consistent with the putative dimer identified in other experiments was visible in H460 cells. We observed that cellular ORF1p and ORF2p levels are not proportional, an observation consistent with the fact that each possesses its own internal ribosome entry site (IRES) [4].

Constitutive LINE-1 mRNA expression across these cell lines (Figure 7C) was measured by One-Step RT-qPCR and normalized to β-Actin (as opposed to the RTC), as cellular LINE-1 mRNA was abundant and its measurement is unaffected by residual genomic DNA levels. A549, H460, and BEAS2B all exhibited low levels of LINE-1 mRNA expression, between 0.27 and 0.38 FC from β-Actin. This was followed by H441 and HCC827, which exhibited 0.79 and 0.81 FC, respectively. H1299 expressed LINE-1 mRNA at 1.10-fold from β-Actin, while H520 cell the exhibited highest expression levels at 5.52-fold above β-Actin expression.

We then measured LINE-1 EV content and EV RT activity from three independent cultures and EV collections (Figure 7D; minimum, maximum, mean shown). EVs were isolated using PEG precipitation and LINE-1 measured using ELISA and One-Step RT-qPCR. The mean RT activity of all six plasma donors from Figure 6F (0.03 ng) is shown for comparison (“Plasma EVs”). BEAS-2B, the non-cancer cell line, exhibited the lowest mean RT activity of negative 0.03 ng. A549 and H460 EVs exhibited residual activity, generating an average of 0.14 ng nucleic acid. H520 EVs generated 0.35 ng, followed by H441 generating 0.81 ng. H827 had a mean of 1.41 ng. H1299 exhibited the highest activity, generating 2.14 ng of material. We noted that RT activity was lowest in the plasma of healthy human subjects and the normal lung epithelial cell line compared to the cancer cell lines, although these observations must be couched in the fact that there are major differences in source material between plasma and cell culture EVs.

As LINE-1 RT activity requires the formation of a ribonucleoprotein comprised of ORF1p, ORF2p, and LINE-1 mRNA, we questioned whether EV levels of these constituents were associated with EV RT activity. If so, this would support the hypothesis that EV RT activity is driven by LINE-1, and provide insight into which constituent might be the most influential. EV LINE-1 ORF1p levels were measured using ELISA and results were normalized to A549 cells, which had the lowest ORF1p. EV LINE-1 mRNA was quantified as previously described and also normalized to A549 cells for consistency. We did not have enough sensitivity to measure ORF2p in EVs. Using simple linear regression, we observed a significant relationship between RT activity and EV LINE-1 mRNA abundance (Figure 7F; R^2^ = 0.81; *P* = 0.006) but not ORF1p levels (Figure 7E; *p* = 0.56). As EV LINE-1 cargo contents mirror their cellular levels (manuscript in preparation), we provided a comparison of cellular ORF2p measures and RT activity to approximate this relationship (Figure 7G), which approached significance (*p* = 0.09). These data suggest that EV LINE-1 mRNA and perhaps ORF2p are most influential over LINE-1 EV RT activity.

## 3. Discussion

The recent explosion of EV research has uncovered a relatively unexplored facet of LINE-1 biology. Initial reports have documented enzymatically active LINE-1 activity in EVs capable of modifying the genomes of recipient cells [17,18]. Given the role of LINE-1 as both an initiator and mediator of various cancers, these findings raise important questions regarding the role of EV LINE-1 in metastatic processes and its potential utility as a cancer biomarker. However, before advances can occur in these areas, a foundation of LINE-1 EV biology must be established. With this goal in mind, the present study characterized the export of LINE-1 cargo in EVs and examined the functionality of the LINE-1 machinery in discrete EV populations.

Using density gradient ultracentrifugation, we confirmed previous reports that LINE-1 mRNA is present in EVs [17,18]. Moreover, we documented for the first time the presence of endogenous ORF1p within EVs. ORF1p has a coiled-coil domain that facilitates the formation of ORF1p multimers that are resistant to SDS-PAGE [26,28]. ORF1p was detected in EVs as a dimer, in sharp contrast to cell lysates where it is predominantly found in the monomeric form. While ORF1p dimers may represent partially denatured trimers [29], our data argue against this interpretation given that lysates from cells and EVs were subjected to identical experimental conditions and run on the same gels. These findings suggest that protein-protein interactions, processing, or packaging differentiates the ORF1p found in cells versus EVs.

When we examined the distribution of LINE-1 mRNA and ORF1p within EV populations, we noted a conserved pattern both in vitro and in vivo. We observed two types of LINE-1-containing EV fractions: small, low-density EVs containing predominantly LINE-1 mRNA and larger medium/high density fractions containing LINE-1 mRNA and ORF1p. The fact that RT activity is present in the same fractions that contain both LINE-1 mRNA and ORF1p suggests that ORF2p may be present in those fractions as well. Other studies have found that low-density and high-density EVs differ substantially with respect to their biophysical properties, proteomic profiles, and RNA content [30,31]. As such, the observed differences in density, size, and composition are likely due to differences in EV biogenesis and cargo loading [23,32]. A remaining question is how LINE-1 is packaged within EV fractions containing multiple LINE-1 components, i.e., are they packaged as a ribonucleoprotein in the same vesicle, or packaged separately in vesicles of similar size and density? A major challenge in addressing this question is that endogenous ORF2p is notoriously difficult to detect and evade even highly sensitive quantification methods such as mass spectrometry [33]. Measuring RT activity is a potential work-around for these direct detection methods; however, it is possible that other endogenous retroelements, such as human endogenous retroviruses (HERVs), could contribute to RT activity.

Using a LINE-1 reporter cell line, Kawamura et al. (2019) demonstrated that EVs can deliver enzymatically active LINE-1 to recipient cells that can subsequently modify host genomes [18]. Given the ability for these EVs to make potentially disruptive changes to the genome, it is possible that RT activity in EVs is related to cancer risk. Indeed, our cell panel investigation revealed that EVs from cancer cell lines exhibited 5–85 times the RT activity from the non-cancerous cell line EVs and plasma EVs from healthy donors. Based on these initial observations, future studies implementing a large number of both normal and cancerous cell lines will be required to further investigate EV RT activity as a function of cancer status and tissue type. One drawback of this assay is that it is somewhat of a ‘black box’, providing a single readout that is likely influenced by many different variables, specifically stochiometric levels of EV LINE-1 mRNA, ORF1p, and ORF2p. Moreover, it is possible that other elements, such as HERVs, contribute to EV RT activity.

To support the hypothesis that EV RT activity is predominantly a readout of LINE-1 RT, we compared EV RT activity with the quantities of individual LINE-1 molecules, using cellular ORF2p levels as a proxy of EV ORF2p content. The abundance of EV LINE-1 mRNA was highly correlated with EV RT activity, with cellular ORF2p levels approaching significance. We noted that the EVs with the highest RT activity levels (H1299, H520, and HCC827) also exhibited moderate/high levels of all three LINE-1 components (positive for cellular ORF2, abundant LINE-1 mRNA, and ORF1p) and that the cell lines with low/absent RT activity were missing at least one of these components. We could not create a multiple linear regression model relating all three components to RT activity, likely due to the small size of the panel. However, the strength of the relationship between RT activity and EV LINE-1 mRNA supports the hypothesis that LINE-1 is the main driver of reverse transcription in EVs.

When we assessed LINE-1 levels within plasma EVs, we observed that ORF1p was present in all donors but that the expression pattern exhibited considerable variability. Plasma EV ORF1p likely originates from tissues that have endogenous expression of LINE-1, such as the brain [34], esophagus, prostate, stomach, or heart muscle [35]. Conversely, we were only able to detect residual EV LINE-1 mRNA in a few individuals. Our results suggest that RT activity is highly dependent on the amount of EV LINE-1 mRNA; thus, the relative dearth of LINE-1 mRNA could serve as an important mechanism to restrict EV RT activity. Future studies could determine if EV LINE-1 mRNA is elevated in individuals with elevated cancer risk, active tumor growth, or metastatic disease.

In conclusion, the findings in this study provide important foundational knowledge regarding the intersection between LINE-1 and EV biology. Moreover, we demonstrate that LINE-1 content and RT activity can be measured in volumes of plasma that are easily obtained from patients during routine venipuncture and widely available from biorepositories. Together, these data may aid the development of a LINE-1-based liquid biopsy that could be used to non-invasively monitor oncogenic progression.

## 4. Materials and Methods

### 4.1. Tissue Culture

Cell lines were purchased from the American Type Culture Collection (ATCC, Manassas, VA, USA) and confirmed to be free of mycoplasma contamination [24]. All cell lines were cultured in a humidified environment at 37 °C, 5% CO_2_ and seeded to 70% confluence for 24 h. BEAS-2B cells were cultured in LHC-9 medium, A549 cells in DMEM plus 10% FBS, and the remaining lines in RPMI plus 10% FBS (Gibco/ThermoFisher Scientific, Waltham, MA, USA). Prior to EV collection, cells were washed with DPBS and incubated in their respective media for 48 h and using EV-depleted FBS (Gibco/ThermoFisher Scientific) as required. Conditioned media were collected and processed for EV isolation. To study LINE-1 inducible conditions, 2 × 10^6^ H460 cells were seeded in 150-mm dishes and allowed to attach overnight. Cells were washed with DPBS and incubated with RPMI medium containing 10% EV-depleted FBS plus 1 μM BaP dissolved in 0.2% DMSO. Conditioned media were collected after 48 h and processed for EV isolation.

### 4.2. EV Isolation from Cell Media

Approximately 120 mL of conditioned media was centrifuged for 5 min at 500× *g* and concentrated to <12 mL using Centricon Plus-70 100 kDa MWCO centrifugal filter units (Millipore-Sigma, Burlington, MA, USA). The concentrate was centrifuged at 21,800× *g* for 30 min to pellet cellular debris and large apoptotic bodies. EVs were isolated using Polyethylene Glycol (PEG), ultracentrifugation, or density gradient ultracentrifugation, as indicated.

For PEG isolation, PEG -MW 6000 (Millipore-Sigma, Burlington, MA, USA) was added to the supernatant to 10% *w/v*, incubated on ice for 30 min, and centrifuged at 21,800× *g* for 30 min. To remove protein contaminants, the EV pellet was resuspended in cold PBS, transferred to a clean tube, and reprecipitated in 10% PEG. For ultracentrifugation, the supernatant was ultracentrifuged at 100,000× *g* for 2 h at 4 °C using a Beckman SW41Ti rotor. The EV pellet was resuspended in 11 mL cold PBS and the ultracentrifugation was repeated. For buoyant density gradient ultracentrifugation, the cleared supernatant was ultracentrifuged once, as previously described. EV pellets were then loaded into the bottom of an ultracentrifuge tube and various iodixanol solutions diluted with DMEM were layered on top according to the following: 3 mL each of 40%, 20%, 10%, and 6%. Following 18 h of ultracentrifugation at 100,000× *g* 4 °C, fractions were removed 1 mL at a time and density was assessed using refractometry. Fractions were diluted with 11 mL cold PBS and ultracentrifuged for 2 h, after which pellets were resuspended in cold PBS. In all experiments, EV abundance was quantified by protein measurement via absorbance at 280 nm. Total EV protein levels were used to normalize EV input across experimental conditions.

### 4.3. EV Isolation from Human Plasma

Human plasma was purchased from BioIVT (Westbury, NY, USA) from ostensibly healthy subjects without serious medical conditions. Blood was collected using IRB-approved protocols and written informed consent was obtained from all subjects. Plasma was collected in K_2_EDTA vacutainer tubes. Upon thawing, platelets were removed after two centrifugation cycles at 3600× *g*. Cleared plasma was diluted 5× with DPBS and centrifuged at 21,800× *g* for 30 min to pellet debris. The supernatant was ultracentrifuged as described above. EV abundance was quantified at 280 nM. Protein was used to normalize EV input.

### 4.4. Nanoparticle Tracking Analysis (NTA) and Transmission Electron Microscopy (TEM)

EV pellet diameter and quantity were assessed using Nanoparticle Tracking Analysis on the Nanosight and ZetaView Instruments (Nanosight, Malvern Panalytical, Malvern, UK and ZetaView, Particle Metrix, Meerbusch, Germany). Nanosight NTA of cell media preparations was performed by the Center for Nanotechnology in Drug Delivery at the University of North Carolina, Chapel Hill (Chapel Hill, North Carolina, USA). For NTA, EVs were diluted in PBS and each sample was read 5–10 times. TEM was performed by the University of Arizona Imaging Core (Tucson, AZ, USA). Discharged carbon-coated grids were floated face-down on a drop of exosome suspension for 4 min. Excess liquid was drained and grids were re-floated on a drop of 1% phosphotungstic acid for 2 min, drained of excess, and air-dried. Grids were viewed in a T12 BioTwin electron microscope.

### 4.5. Western Blotting

Cells and EVs were lysed in RIPA buffer and subjected to Western blotting as previously described [36], with modifications as noted for ORF2p. Immunoblots were imaged using a Konica Minolta SRX101A film developer and the Licor Odyssey FC Imaging System (LI-COR Biosciences, Lincoln, Nebraska, USA). The ORF1p polyclonal antibody was custom-made using the 14-amino acid N-terminus of the ORF1p protein (ORF1p1-14). The specificity of this antibody has been confirmed in previous studies [37]. Antibodies to ALIX (92,880), Flotillin-1 (18,634), and GAPDH (2118) were purchased commercially from Cell Signaling Technology (Danvers, MA, USA). ORF2p immunoblotting was performed using an overnight tank transfer at 21V. The ORF2p antibody was developed by the laboratory of Dr. Belancio [38] and purchased from Kerafast (ETL005).

### 4.6. ELISA

As SDS-PAGE is incompatible with EV PEG isolation [39], ORF1p was measured using a competitive indirect ELISA, as indicated. Briefly, pre-blocked streptavidin-coated plates (Pierce/ThermoFisher, Waltham, MA, USA) were incubated with biotinylated ORF1p1-14. After washing with PBS/0.01% Tween, diluted plasma, standards, and primary antibody were mixed, added to the plate, and incubated for 1h. Standards were generated using known quantities of ORF1p1-14 and a matrix control of EV pellets derived from murine cells. After washing and incubation with HRP-linked secondary antibody (ThermoFisher), the ELISA was developed using TMB (Pierce/ThermoFisher, Waltham, MA, USA) substrate and subsequently quenched with sulfuric acid. Absorbance was read at 450 nM.

### 4.7. LINE-1 mRNA Quantification

EV protein was used to normalize EV input across cell lines and treatments. RNA was extracted from EV pellets using a Quick RNA kit (Zymo, Irvine, CA, USA) with DNAseI digestion (Turbo DNA Free, Invitrogen/ThermoFisher). RNA was eluted in equal volumes of nuclease free water, and 3 μL was in used duplicate reactions of One-Step RT-qPCR, which enables more sensitive detection of less abundant transcripts (Luna One-Step RT-qPCR Kit, NEB, Ipswich, MA, USA).

As LINE-1 contains no introns that distinguish between genomic DNA and mRNA-derived cDNA, each LINE-1 sample was normalized to a matched control lacking reverse transcriptase (RTC) to control for residual gDNA contamination. To measure EV LINE-1 mRNA, the fold change (FC) between samples possessing reverse transcriptase and those without a reverse transcriptase (RTC) was calculated using delta-Ct. β-actin was expressed as FC from the non-template control (NTC). Cellular LINE-1 mRNA was expressed as fold change (FC) from β-actin mRNA, as cellular LINE-1 mRNA was abundant and its measurement is not affected by residual genomic DNA. All primers had amplification efficiencies of >90%. LINE-1 primer: Forward 5′ACACCTATTCCAAAATTGACCAC 3, Reverse 5′ TTCCCTCTACACACTGCTTTGA 3′, and probe 5′ TG GAAACTGAACAACCTGCTCCTGA 3′. β-actin primers: Forward 5′ CTGGCACCCAGCACAATG 3′, Reverse 5′ GCCGATCCACACGGAGTACT 3′, Probe: 5′ ATCAAGATCATTGCTCCTCCTGAGCGC 3′.

### 4.8. Reverse Transcriptase Activity

Reverse transcriptase activity was measured using the EnzChek Reverse Transcriptase Assay Kit (ThermoFisher, Waltham, MA, USA) according to the manufacturer’s protocol with modifications. Briefly, EVs (lysed, intact as indicated) were added to polymerization buffer containing dNTPs. The oligo d(T) primer and Poly(A) ribonucleotide templates were not added to the reaction mixture in order to measure the ability of EV-associated reverse transcriptase to use its own mRNA and residual DNA in target site primed reverse transcription, which is how LINE-1 replicates itself and other RNAs. After a 1-h incubation, PicoGreen reagent was added to measure the fluorescence of RNA-DNA heteroduplexes formed during reverse transcription. For each EV sample, fluorescence from the RT reaction was then normalized to a matched control reaction in which RT was inhibited by the addition of 200 mM EDTA. A lambda DNA standard was used to quantify nucleic acid content.

### 4.9. Statistical Analysis

Statistical analyses were performed using GraphPad Prism 8.1.2. A *p*-value of less than 0.05 was considered significant. EV LINE-1 levels were compared between BaP and DMSO control using Welch’s *t*-test. Total EVs were compared using a *t*-test. To examine subtle BaP-induced changes in EV size, total EV numbers were normalized to 5000 particles per reading and each EV size bin was compared between treatments using a two-way ANOVA. A two-way ANOVA was also used to differentiate between LINE-1 mRNA expression in control and BaP-stimulated gradients. To distinguish between experimental noise and positive LINE-1 mRNA detection, we log2-converted the FC values and performed a one-sample *t*-test to determine if they were significantly different from 0 (no detection). ANOVA was used to compare fluorescence between treatments (one-way) and individuals (two-way). The relationship between RT activity and LINE-1 mRNA, ORF1, and cellular ORF2p was assessed using simple linear regression.

## 5. Patents

Bowers, McKay and Ramos filed patent 2019 62/787444 “Diagnostic Analyte to Enrich LINE-1 and Detect LINE-1 Driven Oncologic Disease: Systems and Methods for Characterizing and Treating Disease”.

## Data Availability

The data that support the findings of this study are available from the corresponding author upon reasonable request.
